# Impact of a Sensorimotor Integration and Hyperstimulation Program on Global Motor Skills in Moroccan Children With Autism Spectrum Disorder: Exploratory Clinical Quasi-Experimental Study

**DOI:** 10.2196/65767

**Published:** 2025-03-26

**Authors:** Rachid Touali, Jamal Zerouaoui, El Mahjoub Chakir, Hung Tien Bui, Mario Leone, Maxime Allisse

**Affiliations:** 1 Faculty of Sciences University of Ibn Tofail Kénitra Morocco; 2 Faculty of Medicine and Health Sciences University of Sherbrooke Sherbrooke, QC Canada; 3 Faculty of Physical Activity Sciences University of Sherbrooke Sherbrooke, QC Canada

**Keywords:** classical physical education, children with a neurotypical profile, children with ASD, UQAC-UQAM test battery, University of Québec in Chicoutimi-University of Québec in Montréal, sensorimotor integration, hyperstimulation, Morocco, sensorimotor, integration, motor skill, Moroccan children, Moroccan, children, autism spectrum disorder, ASD, exploratory study, autism, mental health, young, youth, feasibility

## Abstract

**Background:**

Children with autism spectrum disorders (ASDs) often struggle with processing information, which can impact their coordination, balance, and other motor skills. Studies have demonstrated that intervention programs based on sensory integration can enhance motor performance in these children.

**Objective:**

The objective of this study is to evaluate the applicability of a standardized battery of gross motor skill tests for Moroccan children aged 6 to 12 years with ASD. The objective is to assess the potential efficacy of an innovative pedagogical approach focused on sensorimotor integration and hyperstimulation. This approach will be compared to traditional physical education (PE) sessions to determine its feasibility and potential to bridge the developmental gaps in motor skills between children with ASD and those with a neurotypical profile.

**Methods:**

A convenience sample of 14 Moroccan children with ASD aged 6 to 12 years participated in this exploratory study. Children with ASD were divided into an experimental group (n=7) and a control group (n=7) based on age, sex, motor performance, and socioeconomic status. The control group followed the standard PE program, while the experimental group underwent a specialized program combining sensorimotor integration and hyperstimulation for a period of 15 weeks. All participants were classified as level 2 (moderate) on the Autism Severity Rating Scale based on the *Diagnostic and Statistical Manual of Mental Disorders, Fifth Edition, Text Revision* (DSM-5-TR) criteria. Gross motor skills were measured at baseline and after 15 weeks of intervention using the UQAC-UQAM (University of Québec in Chicoutimi-University of Québec in Montréal) test battery protocol, which includes 10 items.

**Results:**

At baseline (T1), no significant difference was observed between the control and experimental groups of children with ASD. Following the 15-week intervention, the group participating in traditional PE showed an overall improvement in motor skills of approximately 14.5%. Conversely, the results of the ASD experimental group suggest a more substantial improvement of 44.5%. Additionally, the experimental group exhibited significant better performance across all motor skill variables compared to the control group (minimum *P* values of <.02) with large effect sizes (>0.80). In this regard, a 2-way repeated measures ANOVA confirms the efficiency of the program implemented within the experimental group, demonstrating significant effects associated with both group and time factors as well as a clinically highly significant group×time interaction across all measured variables (η^2^p>0.14).

**Conclusions:**

The results of this study suggest that the approach that emphasizes sensorimotor integration and management of hyperstimulation was more effective in improving motor skills in this population. However, other more exhaustive studies will need to be carried out in order to be able to more precisely measure the full potential of this approach.

## Introduction

### Background

Autism spectrum disorders (ASDs) constitute a subset of developmental disorders distinguished by disturbances in communication, changes in social interactions, and repetitive and stereotyped behaviors [1,2]. These symptoms typically emerge before the age of 3 years and result in functional impairments impacting various aspects of daily life [3,4]. Due to the absence of universally recognized biological markers for identifying ASD cases, diagnosis primarily involves evaluation by professionals who assess children’s development trajectories to identify potential developmental disorders [2]. Among the myriad of challenges faced by children with ASD, developmental coordination disorders (DCDs) occupy an important place [1]. Despite being relatively common, these motor difficulties have historically been overlooked [5-7]. However, recent research [8-10] sheds light on the significant prevalence of DCD in children with ASD. In fact, DCD, also known as dyspraxia, is a neurodevelopmental disorder that affects motor coordination and skills. Studies have shown that DCD is remarkably prevalent among individuals with ASD, affecting between 34% and 79% of this population. This is significantly higher than the 6% prevalence rate observed in the general population [11]. This high prevalence of DCD in individuals with ASD suggests that a lack of coordination is a common characteristic of the disorder. Motor deficits can present in diverse forms, encompassing compromised gross motor skills such as running; challenges in fine motor skills like handwriting; difficulties in motor planning and execution leading to problems in coordinating movements and sequencing actions seamlessly; and sensory processing issues such as hypersensitivity or hyposensitivity to sensory inputs, which impact balance, coordination, and motor performance [12-14]. As a result, there is widespread recognition that a deficiency in coordination stands as a prominent trait of ASD, frequently concomitant with motor deficits. The potential etiology of these challenges encompasses both genetic and environmental factors [15].

### Motor Skill Impairment

Typically, children with ASD often face challenges in motor skills, especially in coordination, balance, and postural control [16-19]. A recent meta-analysis even suggests that these deficits extend to decreased walking speed compared to their peers with a neurotypical profile [20]. However, the underlying causes of these impairments remain a topic of debate within the scientific community [21-24]. To address these motor challenges and improve skill development, various interventions have been proposed. These interventions draw inspiration from diverse areas, including sports disciplines [25-27], physical conditioning programs [28], sensorimotor approaches [29], and exercises focused on fundamental motor skills like running, jumping, throwing, and catching [30]. Additionally, research has explored interventions targeting perceptual-motor skills [31]. However, the authors themselves acknowledged the limitations of their programs and called for the development of more precise and specialized interventions. While most studies suggest improvements in motor performance, none, to our knowledge, have directly compared the effects of a general intervention program to one specifically tailored to the needs of Moroccan children with ASD. Such a comparison, focusing on their specific motor difficulties, would be a valuable contribution to the field.

### Sensorimotor Integration and Hyperstimulation Approach for Children With ASD

The field of sensory integration, inspired by the work of Ayres and Robbins [32], offers a valuable approach to improve motor skills in children with neurodevelopmental disorders. This approach hinges on the brain’s ability to organize and interpret information received through the senses (sensory integration). It uses 2 main practices: passive unisensory and active multisensory. Passive unisensory practices involve targeted stimulation of a single sense at a time. These interventions, often used to promote relaxation and emotional regulation, can include massage for muscle relaxation and improved circulation and body pressures to calm the nervous system and reduce anxiety, and weighted vests or blankets to provide deep, comforting pressure. The simplicity and calming effect of these techniques make them particularly beneficial for individuals needing to regulate their emotions. The active multisensory approach stands in stark contrast. Here, individuals actively participate in activities that engage multiple senses at once. This approach aims to cultivate a diverse range of skills through tactile activities (exploring textures), proprioceptive activities (understanding body position and movement), and vestibular activities (developing balance and coordination). By actively engaging individuals in these stimulating activities, the active approach fosters a more comprehensive integration of sensory information. This translates to improved motor skills, coordination, and sensory regulation abilities. The emphasis on exploration and interaction with the environment makes active multisensory practices particularly beneficial for children or individuals with sensory processing difficulties.

At the turn of the 1990s, Polatajko et al [33] explored an approach partially similar to the sensorimotor and hyperstimulation method, working with a group of children diagnosed with DCD. However, when comparing their findings with those of other studies using similar protocols, they reported significantly more mixed results. According to the authors, these limited outcomes may be attributed to the considerable heterogeneity among children with DCD. Indeed, various medical conditions can lead to this disorder, despite having distinct etiological origins. In light of this, the research team advocates for more tailored clinical interventions that take into account the specific characteristics and underlying causes of DCD.

Children with ASD often struggle with processing this information, which can impact their coordination, balance, and other motor skills. Studies have demonstrated that intervention programs based on sensory integration can enhance motor performance in children. Notably, improvements have been observed in postural control, gait, coordination, and hand movements [34,35]. For instance, a systematic review of the effectiveness of interventions using a sensory integrative approach confirmed that these programs can significantly positively affect children’s motor skills [36]. The sensorimotor integration and hyperstimulation approach has been used in children with ASD, particularly those aged 2 to 12 years [37]. These interventions are structured to include a variety of sensory and motor exercises, such as balance activities, coordination games involving crossover movements, and sensory activities like manipulating different textures. These activities are designed to stimulate various senses in a controlled manner, helping children better manage their movements and posture.

### Research Gap

Despite the recent surge in research, some countries, like Morocco, lack precise information on the motor skill profile of their population with ASD. Although numerous studies suggest improvements in motor performance through sensory integration [34-36], no studies, to our knowledge, directly compare the effects of a general intervention program with those of a program specifically based on a sensorimotor integration and hyperstimulation approach. This lack of direct comparison underscores the need for further research to determine the relative effectiveness of these different approaches [38]. This gap is crucial as some authors highlight the considerable variation in children’s motor skills across different countries [39,40]. For instance, studies have shown that Chinese children tend to perform better in manual dexterity and balance tasks, while American children excel in throwing and catching tasks [40]. Moreover, regional disparities offer valuable insights into the development of various motor skills within diverse national contexts.

### Objectives

The primary objective of this study is to evaluate the applicability of a standardized battery of gross motor skill tests for Moroccan children aged 6 to 12 years with ASD. This will provide preliminary data and allow for comparison to a group of children with a neurotypical profile matched for age and sex, thereby estimating the discrepancies in motor development between the 2 groups. The secondary objective is to assess the potential efficacy of an innovative pedagogical approach focused on sensorimotor integration and hyperstimulation for Moroccan children with ASD. This approach will be compared to traditional physical education (PE) sessions to determine its feasibility and potential to bridge the developmental gaps in motor skills between children with ASD and peers with a neurotypical profile.

## Methods

### Design

This study was a clinical, quasi-experimental research with a 2-way repeated measures design conducted on a sample of Moroccan children with ASD aged 6 to 12 years.

### Participants

A convenience sample of 14 Moroccan children with ASD aged 6 to 12 (mean 8.8, SD 1.3) years participated in this exploratory study. This age range was selected because motor skills develop particularly well between 6 and 12 years of age, and the chosen test battery has been validated for children of this age group. Children were randomly divided into an experimental group (n=7; mean age 8.4, SD 1.2 years) and a control group (n=7; mean age 8.5, SD 1.2 years) based on age, sex, motor skill performance, and socioeconomic status. All participants were classified by a psychiatrist as level 2 (moderate) on the Autism Severity Rating Scale based on the *Diagnostic and Statistical Manual of Mental Disorders, Fifth Edition, Text Revision* (DSM-5-TR) criteria. This scale considers the level of support required for the child’s interaction and language to function in their environment. Among the recruited children with ASD, the records showed no other conditions, such as attention deficit disorder with or without hyperactivity, intellectual disability, or anxiety. Children with ASD were cared by the specialized association “El Youssr” since they entered school. This institution’s mission is to support children with special needs. It is located in the municipality of Salé, on the outskirts of the capital Rabat, where most of the participating families have a low socioeconomic status. None of the children participated in extracurricular sports activities or were undergoing medical treatment that could influence their motor skills, such as taking psychotropic drugs. In addition, none of the children had a disabling condition that could be exacerbated by physical activity. The proposed program was designed to ensure minimal disruption to the existing educational and individual student plans within the institution.

### Anthropometric Measures

The anthropometric data were collected in the association’s classrooms, which included body mass (BM), body height (BH), BMI, and body surface area (BSA). BM was assessed using a Seca scale model 760 with an accuracy of 0.5 kg. BH was measured with a Seca stadiometer model 214 to the nearest 0.1 cm. All measurements adhered to the guidelines outlined by Lohman et al [41] for standard operating procedures. BMI was calculated using the formula BM (kg)/BH^2^ (m). BSA was estimated using the method suggested by Mosteller [42], which read as follows:

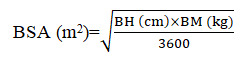



### Motor Skill Assessment

This exploratory study investigated the factors associated with motor skill development in Moroccan children with ASD over a 15-week period. The initial assessment session (T1) established the baseline level of motor skill development in these children. The results from T1 were also used to create control and experimental groups, ensuring no significant difference between them at baseline. Gross motor skills were measured using the UQAC-UQAM (University of Québec in Chicoutimi-University of Québec in Montréal) test battery protocol [43-45]. This battery comprises 13 items assessing five gross motor factors: (1) limb movement speed (upper and lower limbs), (2) agility (circle run, 5-m shuttle run, and side-step run), (3) balance (eyes opened and closed), (4) coordination (ball dribbling and target ball toss), and (5) simple reaction time (SRT; ruler drop test).

For the SRT test, the distance was converted into time (ms) using the equation suggested by Aranha et al [46]:

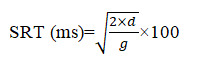



where *d* is the distance (cm) and *g* is the gravitational constant (9.8 m/s^2^).

The choice of this test battery was primarily driven by methodological considerations. First, it assesses a diverse range of motor factors. Additionally, these tests are practical, as they require minimal space, materials, and time and are user-friendly for test administrators, easy for participants to comprehend, and cost-effective, aligning well with the resources of the supervising institution. Given the sensitivity of children with ASD to environmental changes and their challenges with concentration and attention, it was essential to conduct assessments in their classroom settings whenever feasible. The validity and reliability of these tests have been well-established by numerous studies [43,45,47].

### Modified Protocols for the UQAC-UQAM Test Battery

**Figure 1:**
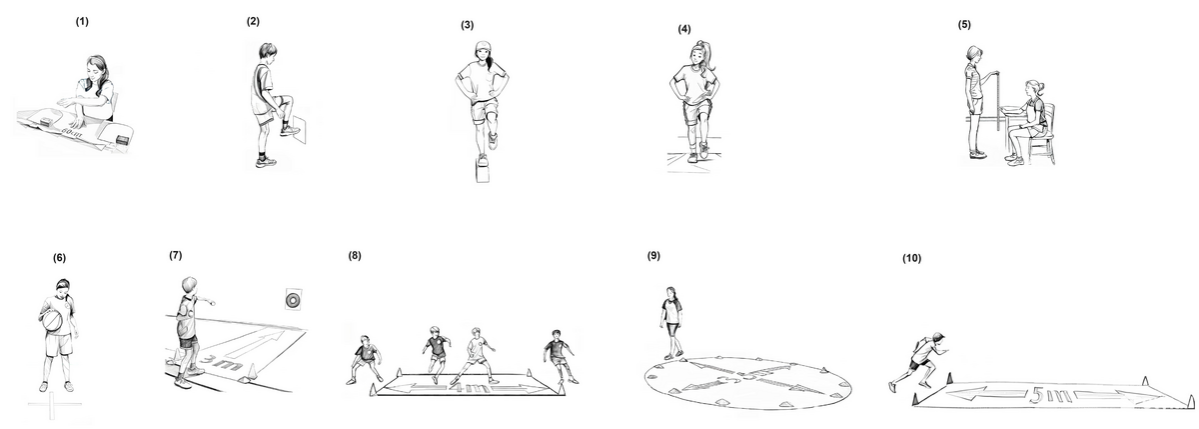
Illustration of the ten tests used to assess motor skill competence in children with ASD: (1) upper limbs speed, (2) lower limbs speed, (3) balance eyes opened, (4) balance eyes closed, (5) simple reaction time, (6) ball dribbling, (7) target ball toss, (8) side-step run, (9) circle run, and (10) 5-m shuttle run (adapted from Allisse et al [49], which is published under Creative Commons Attribution 4.0 International License [50]).

Prior to starting this research project, a week of prospecting was conducted to evaluate the study’s feasibility. Of the original 13 tests within the UQAC-UQAM test battery [43], 3 were omitted due to challenges encountered by the children in comprehending or executing the tasks required (slalom run and hand or foot coordination). Furthermore, 2 tests were adapted to enhance accessibility for children with ASD (Figure 1). The first adaptation concerned on the SRT test. The original version required computer administration, but pilot testing revealed this to be overly complex for the participants. This test was therefore replaced by the procedure suggested by Ángel Latorre-Ron et al [48]. This alternative demonstrated a moderate to good correlation with a computer application (Spearman=0.54; *P*=.03). This change considered the participants’ concentration abilities, as the computerized test was longer than the chosen alternative, which only required 2 attempts. The second adaptation concerned the ball toss test, where the distance between the children and the target was reduced from 5 to 3 m. These modifications to the testing protocol were implemented to ensure the study’s feasibility and to gather reliable and valid data on motor skill development in children with ASD. It was imperative to provide tests adapted to their abilities and limitations to minimize frustration and encourage active and engaged participation.

Following these adjustments, the final test battery comprised the following ten tests: (1) upper limb speed, (2) lower limb speed, (3) balance eyes opened, (4) balance eyes closed, (5) ruler SRT, (6) ball dribbling, (7) target ball toss, (8) side-step run, (9) circle run, and (10) 5-m shuttle run. For the 2 limb speed tests and the ball dribbling test, the score was determined by the greatest number of repetitions within 20 seconds. For the 3 balance tests and the 3 running tests, the score was recorded as time in seconds. In the SRT test, the score was measured in centimeters and then transformed in milliseconds. The target ball toss test score was cumulative, based on the number of points scored in 10 throws (1 point for hitting the target and 2 points for reaching the center). For all tests except the target ball toss, 2 attempts were allowed, and the best of the 2 was recorded as the final score.

### Specific Training Program

The ASD experimental group participated in a specific training program lasting a total of 15 weeks. The program was divided into 3 cycles of 5 weeks each, with each cycle comprising 3 weekly sessions of 45 minutes. This resulted in a total of 45 sessions delivered between November 1, 2021, and April 3, 2022. The pedagogical approach used small group sizes, allowing for individualized monitoring and ensuring a clear understanding of the tasks by participants. The specific training program focused on three key areas: (1) coordination: development of motor skill enabling participants to coordinate whole-body movements smoothly and efficiently; (2) proprioception: enhancement of body awareness and spatial positioning, leading to improved postural control; and (3) agility: strengthening the ability to react quickly and change direction with precision. The intervention process emphasized offering a variety of engaging and playful activities. The overall objective was to provide participants with rich and multimodal sensory experiences that stimulated awareness and intrinsic motivation and presented optimal motor challenges. This included activities such as exercises performed barefoot or with shoes, allowing participants to explore different textures and sensations and exercises using a trampoline to promote body awareness in space and improve balance. Alongside the ASD experimental group, a separate ASD control group participated in regular PE classes based on the standard curriculum as proposed by the Ministry of National Education of Morocco (Table 1). Further details can be found in Multimedia Appendices 1 and 2. The duration and frequency of these classes matched those of the experimental group. The inclusion of an ASD control group allowed for a comparison of motor skill development in participants who underwent the specific training program against those who did not (for a detailed description of both, the intervention programs for the experimental and control groups, please refer to Multimedia Appendices 1 and 2). Furthermore, a neurotypical control group comprising children without ASD was established. This group served as a baseline to evaluate the motor skill level of children with ASD in both the experimental and control groups, taking into account age and locality.

**Table 1:** Detailed comparison of 15-week intervention activities for experimental (sensory intervention) and control (classic intervention) groups in a physical development program for children with autism spectrum disorder.

Block and group		Theme		Content		Operationalization	
**Block 1: 3 times per week (5 weeks)**							
	Experimental	Coordination	Spatial orientation; Execution rhythm and time-space structuring; Segmental differentiation and dissociation; Reaction speed.		Tactile activities: diversifying contact surfaces (barefoot and with shoes); Proprioceptive activities: trampoline and plyometric exercises; Vestibular activities: Swiss ball exercises and orientation; Multimodal sensory experiences tasks; Playful form, optimal motor challenge, and secure and flexible framework; Encourage collaboration and participation.		
	Control	Aerobic fitness	Aerobic capacity (10 to 15 minutes total); Aerobic power (3 to 6 minutes total, various repetitions); Speed (repetitions of 2 to 3 seconds).		Continuous running: interval running (varying long or fast pace); Intermittent: short/short, long/long.		
**Block 2: 3 times per week (5 weeks)**							
	Experimental	Balance	Footwork and body balance mastery; Spatial orientation; Static and dynamic postural balance; Plyometric exercises.		Tactile activities: diversifying contact surfaces (barefoot and with shoes); Proprioceptive activities: trampoline, plyometric exercises; Vestibular activities: Swiss ball exercises, orientation; Multimodal sensory experiences tasks; Playful form, optimal motor challenge, and secure and flexible framework; Encourage collaboration and participation.		
	Control	Strength	Horizontal and vertical jump (explosive strength); Linking running with vertical and horizontal jump; Jump adaptation to space; Movement, acceleration, tension, ejection, and body stabilization; Mastery of principles: slow/fast, back/front, bottom/top, right/left.		Learn phases and sequences of throwing and jumping; Understand general rules of throwing or jumping; Motor organization to jump or throw as high or far as possible.		
**Block 3: 3 times per week (5 weeks)**							
	Experimental	Agility	Linking, braking, and acceleration; Linear and multidirectional speed; Motor flexibility; Body control: adaptation to various stimuli; Rapid and continuous direction changes; Muscle strengthening.		Tactile activities: diversifying contact surfaces (barefoot and with shoes); Proprioceptive activities: trampoline and plyometric exercises; Vestibular activities: Swiss ball exercises and orientation; Multimodal sensory experiences tasks; Playful form, optimal motor challenge, and secure and flexible framework; Encourage collaboration and participation.		
	Control	Cooperation and opposition with ball	Discover a team game: 1 or 2 key rules; Differentiate partner and opponent; Respect spatial boundaries; Discover a target to defend and attack.		Social interactions and exchanges; Communication and information gathering; Cooperation and mutual aid.		

### Statistical Analysis

The data were summarized using descriptive statistics. Central tendency was characterized by means, and variability was assessed by SD. To assess normality for all variables, the Shapiro-Wilk test was conducted. Group mean comparisons were performed using unpaired 2-tailed *t* tests for the control and experimental groups, while paired 2-tailed *t* tests were used to evaluate differences between measurements at time 1 (T1) and time 2 (T2). Effect sizes (ESs) were calculated using the Cohen *d* formula [51] and interpreted using the following scale: <0.20 (trivial), 0.20-0.49 (small), 0.50-0.79 (medium), and ≥0.80 (large). The main effects of group and time, as well as the group×time interaction, were analyzed using a 2-way repeated measures ANOVA. Partial eta squared (η^2^p) values were reported to indicate ESs, interpreted as follows: <0.01 (trivial), 0.01-0.05 (small), 0.06-0.13 (moderate), and ≥0.14 (large). A significance level of *P*≤.05 was used. All statistical analyses were carried out with SPSS (version 24.0; IBM Corp).

### Ethical Considerations

Details of the project were communicated to parents, students with ASD, and education authorities through a written and explanatory document that each party was required to sign. Thus, by enrolling in this project, the individuals involved acknowledged having provided de facto their written informed consent. However, it was agreed that participants could withdraw at any time without penalty or prejudice. The study was conducted in accordance with the Declaration of Helsinki and approved by the institutional ethics committee of Ibn Tofail University (protocol code: 20019741; date of approval: March 3, 2023) for studies involving humans. Data collection only began upon receiving authorization from the presidents of the 2 participating associations. All collected data were anonymized to ensure participant confidentiality and were stored securely in compliance with ethical guidelines. Participants or their families received no financial or material compensation for their participation in this study. This paper does not contain any images or documents that could identify individual participants.

## Results

Table 2 presents the anthropometric characteristics between the ASD experimental group to the ASD control group at baseline (T1). The results show that there are no significant differences in the anthropometric characteristics measured between the children in the experimental group and those in the control group at baseline. This suggests that the 2 groups are well balanced in terms of age, BM, BH, BMI, and BSA, which is crucial to ensure that any future intervention can be evaluated without bias related to initial anthropometric differences between the groups. The *P* values indicate that all comparisons are nonsignificant, with values well above the conventional significance limit of ≤.05, reinforcing the idea of homogeneity between the groups at the start of the study.

**Table 2:** Anthropometric characteristics of children with autism spectrum disorder according to group membership at baseline (T1).

Variables	Experimental group (n=7)		Control group (n=7)		*P* value^a^
	Mean (SD)	95% CI	Mean (SD)	95% CI	
Age (years)	8.5 (1.2)	7.3-9.5	9.1 (1.4)	7.9-10.5	.29
Body mass (kg)	31.0 (7.3)	24.3-37.8	32.7 (8.0)	25.3-40.1	.68
Body height (cm)	131.7 (7.8)	124.5-138.9	133.7 (7.3)	126.9-140.5	.63
BMI (kg/m^2^)	17.9 (3.5)	14.6-21.1	18.1 (2.5)	15.8-20.4	.88
Body surface area (m^2^)	1.06 (0.14)	0.93-1.19	1.10 (0.16)	0.95-1.25	.65

^a^Significant at ≤.05.

Table 3 presents a comprehensive comparative analysis of the motor skill performance evolution among children with ASD, spanning from the initial baseline (T1) to the postintervention period (T2). The data are categorized into 2 groups: an ASD experimental group and an ASD control group. In the ASD control group, significant enhancements were observed in upper and lower limb speed, along with ball dribbling and 5-m shuttle run after 15 weeks in the classic PE program. However, there were no significant differences noted in balance duration with eyes opened and closed. Similarly, variables related to running and coordination, such as circle run, side-step run, 5-m shuttle run, target ball toss, and ball dribbling, did not exhibit significant improvements. Regarding the ASD experimental group, noteworthy advancements were recorded across all variables after 15 weeks in the sensorimotor integration and hyperstimulation program. Examining the overarching changes in motor performance between T1 and T2, a distinct contrast emerges between the 2 ASD groups. The ASD control group witnessed an overall progression of 14.5% across all evaluated motor skill variables. Conversely, the ASD experimental group demonstrated a notably higher progression, reaching 44.5%.

**Table 3:** Comparison of motor skill improvements from baseline (T1) to postintervention (T2) in autism spectrum disorder (ASD) control and experimental groups^a^.

Variables			T1, mean (SD)		T2, mean (SD)		*P* value^b^		Cohen *d* effect size^c^		∆ T1 vs T2 (%)
**ASD experimental group**											
	Upper limb speed (nb/20 seconds^d^)	23.7 (6.3)		37.7 (4.2)		.001		2.12		14.0 (37.1)	
	Lower limb speed (nb/20 seconds)	9.0 (2.1)		16.4 (2.2)		<.001		6.74		7.4 (45.1)	
	Balance eyes opened (seconds)	6.1 (2.6)		14.6 (8.7)		.02		1.21		8.5 (58.2)	
	Balance eyes closed (seconds)	2.3 (0.9)		6.4 (2.0)		.001		1.91		4.1 (64.1)	
	Ball dribbling (nb/20 seconds)	5.3 (4.0)		22.6 (10.9)		.003		1.88		17.3 (76.6)	
	Target ball toss (points)	3.1 (2.2)		8.1 (3.1)		.01		0.89		5.0 (61.7)	
	Side-step run (seconds)	20.4 (3.7)		13.9 (0.9)		.003		1.61		6.5 (31.9)	
	Circle run (seconds)	40.0 (5.8)		28.1 (1.1)		.002		1.13		11.9 (30)	
	5-m shuttle run (seconds)	18.4 (0.8)		15.4 (1.1)		.001		2.15		3.0 (16.3)	
	Simple reaction time (ms)	299.5 (45.6)		223.4 (24.6)		.002		1.78		76.1 (25.4)	
	Mean (SD)	—^e^		—		—		2.14 (1.67)		44.5 (19.8)	
**ASD control group**											
	Upper limb speed (nb/20 seconds)	22.9 (4.9)		27.7 (4.7)		.01		1.45		4.8 (17.3)	
	Lower limb speed (nb/20 seconds)	6.9 (2.0)		10.9 (2.6)		.002		2.28		4.0 (36.7)	
	Balance eyes opened (seconds)	3.2 (1.2)		4.6 (2.6)		.28		0.29		1.4 (30.4)	
	Balance eyes closed (seconds)	1.9 (0.9)		1.9 (1.3)		.91		0.00		0.0 (0)	
	Ball dribbling (nb/20 seconds)	3.0 (0.8)		6.7 (3.4)		.01		3.30		3.7 (55.2)	
	Target ball toss (points)	1.7 (1.9)		1.6 (1.4)		.79		0.10		–0.1 (–5.9)	
	Side-step run (seconds)	21.6 (2.9)		20.5 (4.1)		.25		0.74		1.1 (5.1)	
	Circle run (seconds)	39.9 (7.1)		37.2 (5.6)		.06		2.12		2.7 (6.8)	
	5-m shuttle run (seconds)	18.9 (3.7)		20.6 (3.2)		.007		4.57		–1.7 (–8.3)	
	Simple reaction time (ms)	336.2 (33.2)		310.6 (26.9)		.14		0.67		25.6 (7.6)	
	Mean (SD) (%)	—		—		—		1.55 (1.51)		14.5 (20.4)	

^a^Intergroup differences were measured using a 2-tailed *t* test for unpaired values. Changes between T1 and T2 for each group were calculated using a 2-tailed *t* test for paired values.

^b^Significant at *P*≤.05.

^c^<0.20=marginal; 0.20-0.49=small; 0.50-0.79=moderate; and ≥0.80=large.

^d^nb/20 seconds=number of repetitions in 20 seconds.

^e^Not applicable.

The results outlined in Table 4 reveal significant effects of group, time, and group×time interactions for the majority of gross motor skills. Variables such as segmental speed, balance, ball dribbling, and SRT exhibit the most substantial effects. While improvements are also noted in side-step running and circle running, these gains are comparatively modest. Mauchly test of sphericity indicated that the assumption of sphericity was not violated for any of the dependent variables, suggesting that the variances of the differences between levels of the repeated measures factor were approximately equal. Thus, these findings demonstrate that the intervention was highly effective in enhancing motor performance within the experimental group.

**Table 4:** Summary of a 2-way ANOVA with repeated measures evaluating the effects of group, time, and group×time interaction on gross motor skills performance between the experimental and control groups of children with autism spectrum disorder^a^.

Variables and source		Sum of squares	*df*	Mean square	*F* test (*df*=12)^b^	*P* value^c^	η^2^p^d^
**Upper limb speed (nb/20 seconds^e^)**							
	Group	206.3	1	206.3	5.361	.04	0.309
	Time	622.3	1	622.3	48.044	<.001	0.800
	Group×time	146.3	1	146.3	11.294	.006	0.485
**Lower limb speed (nb/20 seconds)**							
	Group	104.1	1	104.1	12.941	.004	0.519
	Time	228.6	1	228.6	145.455	<.001	0.924
	Group×time	20.6	1	20.6	13.091	.004	0.522
**Balance eyes opened (seconds)**							
	Group	365.6	1	365.6	19.393	.001	0.618
	Time	260.7	1	260.7	33.560	<.001	0.737
	Group×time	133.7	1	133.7	17.206	.001	0.589
**Balance eyes closed (seconds)**							
	Group	42.9	1	42.9	21.122	.001	0.638
	Time	30.6	1	30.6	20.113	.001	0.626
	Group×time	28.5	1	28.5	18.761	.001	0.610
**Ball dribble (nb/20 seconds)**							
	Group	576.0	1	576.0	11.576	.005	0.491
	Time	771.8	1	771.8	31.998	<.001	0.727
	Group×time	322.3	1	322.3	13.364	.003	0.527
**Target ball toss (points)**							
	Group	112.0	1	112.0	18.556	.001	0.607
	Time	41.3	1	41.3	10.671	.007	0.471
	Group×time	46.3	1	46.3	11.963	.005	0.499
**Side-step run (seconds)**							
	Group	106.4	1	106.4	7.020	.21	0.369
	Time	103.4	1	103.4	22.549	<.001	0.653
	Group×time	50.5	1	50.5	11.004	.006	0.478
**Circle run (seconds)**							
	Group	144.3	1	144.3	3.142	.10	0.207
	Time	372.7	1	372.7	30.427	<.001	0.717
	Group×time	148.9	1	148.9	12.151	.004	0.503
**5-m shuttle run (seconds)**							
	Group	56.3	1	56.3	4.669	.05	0.280
	Time	2.5	1	2.5	3.550	.08	0.228
	Group×time	39.1	1	39.1	56.465	<.001	0.825
**Simple reaction time (ms)**							
	Group	26,859.3	1	26,859.3	18.060	.001	0.601
	Time	18,105.4	1	18,105.4	28.592	<.001	0.704
	Group×time	4450.7	1	4450.7	7.028	.02	0.369

^a^Mauchly sphericity test≥0.05 for all the variables in the analysis.

^b^*F*=variance between groups/variance within groups.

^c^Significant if ≤.05.

^d^Small effect size: η^2^p=0.01; moderate effect size: η^2^p=0.06; and large effect size: η^2^p=0.14.

^e^nb/20 seconds=number of repetitions in 20 seconds.

## Discussion

### Principal Findings

Autism, a complex spectrum of neurodevelopmental conditions, is characterized by a wide range of manifestations and challenges. Recent research, such as that conducted by Khoury et al [52], has shed light on a connection between sensorimotor integration disorders and ASD. These disorders impact both fine and gross motor skills, as stated by some studies that identified 2 primary anomalies contributing to the motor difficulties observed in individuals with ASD, which are impaired integration of information crucial for motor planning and heightened variability in basic sensory input and output [24,53].

### Characteristics of Motor Performance in Individuals With ASD

Unlike individuals with a neurotypical profile, who readily integrate information from multiple senses (multimodal integration), people with ASD often process this information independently for each sense (unimodal integration). This unique characteristic can lead to difficulties in perception when exposed to simultaneous stimulation across multiple senses. As part of a study comparing the motor performances of individuals with and without ASD, the battery of tests proposed by Leone et al [43,44] proved to be a relevant tool. First, this battery effectively evaluates a broad range of fundamental motor skill factors (segmental speed, coordination, balance, agility, and reaction time), which was important in the context of this study. Second, the test items are designed as simple, isolated tasks, minimizing the influence of other motor components. This structure aimed to reduce potential performance discrepancies between the 2 participant groups. However, despite these precautions, the study revealed significant differences in motor performance between children with a neurotypical profile and those with ASD from the outset. Children with ASD demonstrated a marked delay in motor development, even in tasks primarily reliant on unimodal sensory processing.

### The Classic Versus the Sensorimotor Integration and Hyperstimulation Approach

In efforts to integrate children with ASD into the education system, it is common for PE lessons to be taught using a classical method similar to that used for children with a neurotypical profile. Morocco follows this trend and offers children with autism the same PE program prescribed by the Ministry of National Education. Although some adaptations are implemented, such as reducing class sizes, this type of program, which is based on disparate and eclectic exercises derived from sports activities, does not sufficiently consider the unique characteristics of children with autism. This approach results in a significant increase in the quantity and variety of sensory and motor stimulation, which conflicts with the typical functioning of children with autism. Additionally, these children often experience sensory hyperreactivity or hyporeactivity to environmental stimuli such as brightness, ground texture, and ambient noise. This can disrupt their routine and, consequently, hinder their learning capacity [54]. Despite their limitations, traditional PE classes can demonstrably contribute to improve some motor skills in children with ASD. Our study showed that a 15-week classical PE program allowed a nearly 14.5% overall improvement in the control group of children with ASD. This finding highlights the capacity of traditional PE methods to induce, to a certain extent, a contribution to the motor development of this population.

In contrast, the experimental group, whose PE lessons were adapted based on principles of sensorimotor integration and hyperstimulation management, demonstrated a remarkable 44.5% improvement over the same 15-week period. Aware of the debates that this approach still generates, we have introduced innovations that, in our opinion, have contributed to this impressive outcome. While sensorimotor integration appears more appropriate for children with ASD than traditional methods, most research advocates for a unimodal approach, emphasizing tasks that engage a single sense. Although some studies show promising results, others question the effectiveness of this method, suggesting that it fails to adequately address the specific needs of children with ASD.

In this study, a multimodal approach was adopted. Each block focuses on a specific single motor skill in a manner that engages multiple senses simultaneously. For instance, while block 1 emphasizes coordination, learning situations necessitate the involvement of various senses to accomplish each task. Furthermore, to control for environmental variables and minimize external influences, all sessions were conducted in a single room, ensuring consistent lighting, playing surface, and ambient sound when the learning situation requires it. Given its unique characteristics, the program structure developed in this study represents a valuable contribution to the field.

### ASD Experimental Versus Control Group

The results of this study are highly encouraging and suggest that the implemented intervention had a significant impact on improving the motor skills of children with ASD. Indeed, all motor skill factors assessed showed significant improvement in the experimental group compared to the control group (*P*<.05). The enhancements were not only statistically significant but also clinically relevant, as indicated by large ESs (Cohen ES>0.80). These findings support the idea that targeted interventions can enhance motor skills in children with ASD, with potential broad-ranging implications for their overall development.

The results of the 2-way repeated measures ANOVA strongly support the effectiveness of the sensorimotor integration and hyperstimulation method. The findings reveal that the group×time interaction is significant for several variables, indicating that performance varies not only by group but also over time. This interaction suggests that the effect of time on motor performance is not consistent across groups. In other words, the interventions or experimental conditions had different impacts depending on group membership. It is therefore likely that the experimental group showed more substantial improvements over time compared to the control group, which could suggest greater effectiveness of the implemented intervention. The ESs (η^2^p) associated with these interactions are also noteworthy. The high ESs further emphasize that the intervention had a significant clinical impact. These findings emphasize the importance of individualized training programs that consider specific needs and learning styles to optimize motor development outcomes.

The observed improvements spanned a wide range of motor domains, including coordination, balance, and reaction time, suggesting a comprehensive enhancement of motor skills. This study highlights the importance of incorporating specific physical activities into educational programs for children with ASD. By promoting motor skill development, one can significantly improve these children’s quality of life, learning, and social inclusion. Moreover, this study raises intriguing questions regarding the underlying neurobiological mechanisms that may account for the observed improvements in children with ASD following the intervention. While further research is needed to draw definitive conclusions, several potential mechanisms could be explored.

### Potential Neurobiological Mechanisms Underlying Motor Skill Improvements in Children With ASD

Regular physical exercise stimulates the production of neuronal growth factors, which promote the formation of new synaptic connections in brain regions involved in motor control. Training can lead to the reorganization of neural networks, enhancing movement coordination and motor planning [55]. Physical activity is linked to dopamine release, a neurotransmitter integral to reward systems. This could boost children’s motivation to engage in physical activities and reinforce adaptive motor behaviors. Dopamine also plays a pivotal role in motor control, and its modulation could contribute to the observed improvements [56]. Additionally, serotonin, which regulates mood and anxiety, may see increased activity due to physical exercise, potentially reducing the anxiety symptoms often associated with ASD and facilitating the acquisition of new skills [57]. Physical exercise can enhance sensory perception, including proprioception and vestibular sense, thereby improving movement coordination [58]. Our proposed sensorimotor integration and hyperstimulation program specifically addresses these 2 sensory modalities. Although these hypotheses are plausible, further research is needed to assess their validity.

### Implementation of a Sensorimotor Integration Program in the Moroccan Context

Similar to many societies, Morocco often prioritizes the classical PE approach to stimulate motor development in children, including those with ASD. However, the findings of this exploratory study suggest that an alternative approach focused on sensorimotor integration and hyperstimulation management could be particularly beneficial for Moroccan children with ASD. Conducted within the Moroccan cultural and educational context, the study demonstrated that this innovative approach could not only be successfully implemented but also yield significantly better results in motor development compared to the classical approach. While traditional PE classes can offer some benefits to children with ASD, integrating principles of sensorimotor integration and hyperstimulation management into PE programs can lead to more substantial improvements. The use of a multimodal approach, where tasks are designed to engage multiple senses, appears to be particularly effective. This method not only addresses the unique sensory processing challenges faced by children with ASD but also helps to bridge the developmental gap between them and their peers with a neurotypical profile. As such, the program developed in this study represents a significant advancement in the field of PE for children with ASD, providing a framework for future research and practice.

### Limitations and Strengths

This study reveals several limitations. The small number of participants with ASD (n=14) requires caution in interpreting the results. Additionally, the fact that the age range is limited to 6 to 12 years makes the results potentially inapplicable to other age groups. Given that these children mainly come from disadvantaged backgrounds, it is uncertain whether the same findings would be observed in different socioeconomic contexts. All participants with ASD were classified as level 2 (moderate) on the Autism Severity Rating Scale according to DSM-5-TR criteria, so it is not possible to judge whether the program would have the same effectiveness for severity levels 1 and 3. However, this limitation ensures that the group of children with ASD was homogeneous, which is often a weakness in other studies. Additionally, obtaining repeated measurements allows changes to be better assessed since they come from the same children at different time periods. This study also has the advantage of providing, for the first time, a preliminary portrait of the motor development of Moroccan children with a neurotypical profile versus children with ASD based on the UQAC-UQAM test battery. The proposed approach (sensorimotor integration and hyperstimulation), as implemented in this study, is also a first for the Moroccan population with ASD.

### Conclusions

Children with ASD often exhibit a unimodal sensory processing style, meaning they tend to focus on one sensory input at a time. This can lead to significant perceptual challenges when they are exposed to stimulation that engages multiple senses simultaneously. Conventional PE programs, which typically use a variety of exercises derived from various sports, may actually hinder the progress of children with ASD by overloading their sensory systems. In contrast, a novel approach that emphasizes sensorimotor integration and hyperstimulation management appears to be much more effective in improving motor skills in this population. This method prioritizes engaging multiple senses concurrently while minimizing excessive sensory stimuli. This study revealed that the sensorimotor integration and hyperstimulation management program yielded a remarkable 44.5% improvement in motor skills for children in the experimental group over a 15-week period. This is in stark contrast to the 14.5% improvement observed in the control group who participated in the traditional PE program. While this study is limited by the sample size, it provides a groundbreaking initial assessment of motor development in Moroccan children with ASD. Furthermore, it proposes an innovative approach specifically tailored to their motor skills condition and the prevailing sociocultural and educational context within Morocco. However, other more exhaustive studies will need to be carried out in order to be able to more precisely measure the full potential of this approach.

## Appendix

Multimedia Appendix 1 The intervention program for the experimental group.

Multimedia Appendix 2 The intervention program for the control group.

## Abbreviations

ASD: autism spectrum disorder
BH: body height
BM: body mass
BSA: body surface area
DCD: developmental coordination disorder
DSM-5-TR: Diagnostic and Statistical Manual of Mental Disorders, Fifth Edition, Text Revision
ES: effect size
PE: physical education
SRT: simple reaction time
UQAC-UQAM: University of Québec in Chicoutimi-University of Québec in Montréal

## Data Availability

The original contributions presented in the study are included in the paper. Further inquiries can be directed to the corresponding author. All data generated or analyzed during this study are included in this published paper.
