# Supplementary material for: Impact of a Sensorimotor Integration and Hyperstimulation Program on Global Motor Skills in Moroccan Children With Autism Spectrum Disorder: Exploratory Clinical Quasi-Experimental Study
Source: JMIR Form Res. 2025 Mar 26;9:e65767. doi: 10.2196/65767 (PMC11982752; doi:10.2196/65767)
Supplement: Multimedia Appendix 1 [file formative_v9i1e65767_app1.pdf]

Cycle 1: Primary dominant proprioception (balance) and secondary dominant coordination						
General (15 min) and specific (5 min) warm-up routine for each session						
**Week**	**Session**	**Sequence**	**Objectifs**	**Exercise description (20 min/exercise including, exercise variations)**<br>*4 to 6 repetitions per exercise variant*	**Exercise(s)**	**#**
1	1, 2, 3	**Motor Skills Assessment (T1)**				
2	4, 5, 6	Control of supports, static and dynamic balance	Be able to maintain balance between each movement	The participant has both feet in a hoop. He/she must place another hoop in front of him and then jump into it with his feet together. He/she must repeat this until the finish line is reached. **Variation:** with a unipodal jump in each hoop, integrate competitions, velocity of execution.	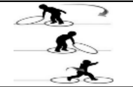	1 a
			Be able to maintain dynamic balance while standing	The participant should pass a ball while standing with both feet in the middle of a hoop. Variations: supports aligned then staggered, with unipodal support. Changing the support surfaces (mat, foam, balance ball, trampoline). Changing the position of the receiver to the right/left. Throwing with eyes open or eyes closed, with or without shoes.	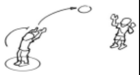	1 b
			Be able to maintain dynamic balance while seated	The child should pass and receive a ball sitting on a Swiss ball with their knees bent at 90° feet and still in the middle of the hoop while the teacher is in front of him/her. **Variation** : Change the orientation of the teacher, feet apart and together, without or with shoes, balls of different textures soft, hard, rough, soft, smooth, wavy, heavy, light.	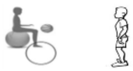	1 c
3	7, 8, 9	Spatial orientation	Be able to run quickly to a specific destination	Four different colors arranged on the 4 directions, the teacher announces the color towards which the child must run and touch it. **Variations:** change the distance between the cones, change the placement of the cones.	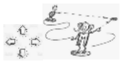	2 a
			Be able to roll a ball following a route	The child must roll a basketball following a route drawn on the ground by a string. Perform three to four repetitions. **Variations:** change the texture and weight of the balls; change the route drawn on the ground.	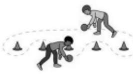	2 b
			Be able to move with a ball and stop in a specific place without losing balance	Holding a ball, the child moves towards a destination bearing a number announced by the teacher, he must be able to stop in a hoop without losing his balance. **Variations**: change the rhythm, number and space between the hoops	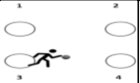	2 c
4	10, 11, 12	Static and dynamic postural balance	Be able to maintain a static balance on an unstable surface	The child balances himself in a stable standing position on a bosu ball. **Variations**: Arms up, arms to the side, unipodal balance for three to four seconds	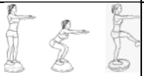	3 a
			Be able to maintain balance while walking on a variety of surfaces	The child must step on objects of different heights and textures without touching the ground while keeping their balance and finishing with a basketball throw. **Variations**: modify the distance.	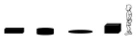	3 b
			Be able to balance objects while walking on unstable surfaces	The child must walk on the rope while holding a bar with two rings on the ends. **Variations**: barefoot, change the route of the rope from straight to winding, change the weight of the rings.	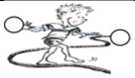	3 c
5	13, 14, 15	Pliometrics	Be able to jump over obstacles and throw a ball with precision.	The child evolves first by walking and then running on a six-meter circuit including three "rivers" to jump, ending with a shot. **Variations**: change the number and width of rivers	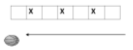	4 a
			Be able to throw after a countermovement jump	The child jumps from a crate, landing feet together in a hoop, then performs a suspended throw on the basketball hoop. **Variations**: replace the hoop with a trampoline, add another hoop, vary the distance of the throw.	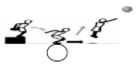	4 b
			Be able to perform a double task in the same action	By jumping on a trampoline, the child receives and passes balls thrown by the teacher. Variations: unipodal support, barefoot, modify the distance between the child and the teacher, change the direction of the shot, add two or three launchers.	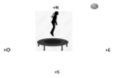	4 c
6	16	Review of achievements and reinforcement		All elements already seen in the cycle are resumed in the form of a course	1a, 1b, 1c, 2a	
	17				2b, 2c, 3a, 3b	
	18				3c, 4a, 4b, 4c	
Cool-down routine (5 min) at each session						

Cycle 2: Coordination under proprioception dominance

General (15 min) and specific (5 min) warm-up routine for each session

Week	Session	Sequence	Objectif	Exercise description (20 min/exercise including, exercise variations)<br>*4 to 6 repetitions per exercise variant*	Exercises	#
7	19, 20, 21			**Motor Skills Assessment (T2)**		
8	22, 23, 24	Spatial orientation	Be able to direct your jumps with precision	The child jumps and alternately puts the right foot in the hoops placed on the right then on the left and then shoots towards the target. **Variations**: add hoops, change the distance between hoops.	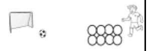	5 a
			Be able to locate oneself in space and shoot a ball	The student crosses an area by chaining jumps on hoops, leaving the area the child shoots at the target. **Variations**: bipodal support and unipodal support, modify the number of hoops vary the orientations of the doors and the hoops.	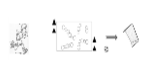	5 b
			Be able to perform jumps in different predetermined axes	The child jumps feet together in hoops respecting the orientation and direction of the arrow, and ends with a shot. **Variations**: space out the hoops, vary the direction of the arrows, unipodal support.	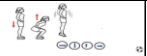	5 c
9	25, 26	Laterality and segmental differentiation	Be able to rotate around a transverse axis to the right and left	Lying on the ground with the ball in hand, the child rolls to the side and places the ball in the basket, once to the right and once to the left, with the basket placed at head level. **Variations**: change the location of the basket, move the basket away.	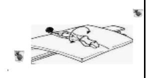	6 a
			Be able to orient yourself to kick or put a ball in a specific place	The child must run on a straight line, go around the cones arranged to the right and left of the line, then kick the ball to return each time to the starting line. **Variations**: modify the distance of the cones, the rhythm of execution, use balls of different textures.	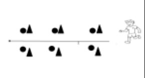	6 b
	27, 28, 29	Rhythm of execution and structuring of time and space	Be able to control the amplitude and frequency of your support	The child must walk, run or jump by placing one foot in each box, then finish with a shot at a target. **Variations**: vary the space between the squares, change the rhythm of passage, bipodal support.	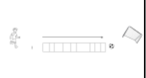	6 c
10			Be able to perform vertical and horizontal jumps to overcome obstacles	The child must jump over "rivers" and step over hurdles, retrieve a ball and return to the starting line to place it in a basket. **Variations**: jumping, alternating horizontal and vertical jumps, changing the distance between obstacles and their height	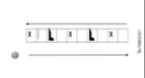	7 a
			Be able to adapt motor actions according to the nature of the space	The child evolves on a course where he steps over hurdles, jumps in hoops (unipodal support), crawls to cross a hoop, and ends with a slalom run. **Variations**: change the order of the exercises and the direction of the course.	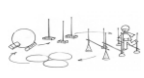	7 b
	30, 31	Segmental differentiation and dissociation	Be able to move around without using your hands	Jump simultaneously feet apart in a red hoop then feet together in the blue hoop while holding an object in both hands then finish with a shot. **Variations**: change the objects carried by hand: volume weight and texture (ball, medicine ball, bar), change of the hoops by a coordination scale with small boxes and another large one.	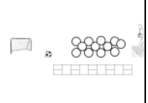	7 c
11			Be able to recover a ball and move in an unusual situation	The teacher rolls balls towards the student in an inverted quadruped position, who must receive and control the ball with their feet. He can then move (quadruped) and put them back in the hoop on the left then on the right**. Variations**: modify the distance of the hoops, the volume and the texture of the balls, same situation but the child is seated on a chair.	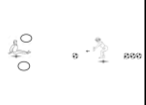	8 a
	32, 33	Response to a signal	Be able to react quickly to a visual signal	The child is behind a line, 3 meters from the teacher who drops a scarf. The child must retrieve it before it hits the ground. **Variations**: plastic bag, change distance, with all students.	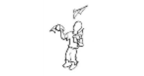	8 b
			Be able to adapt the motor action in different situations	Children are arranged in a circle around the teacher who is spinning a stick of foam at ground level. Children must jump over to avoid being touched. Then, the teacher turns the stick at shoulder height for the children who must avoid it by crouching down. **Variations**: the teacher alternates the direction of the stick.	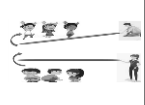	8 c
12	34	Review of achievements and reinforcement		All elements already seen in the cycle are resumed in the form of a course	5a, 5b, 5c, 6a	
	35				6b, 6c, 7a, 7b	
	36				7c, 8a, 8b, 8c	

Cool-down routine (5 min) at each session

Cycle 3: Agility under coordination and proprioception dominant						
Cool-down routine (5 min) at each session						
**Week**	**Session**	**Sequence**	**Objectif**	**Exercise description (20 min/exercise including, exercise variations)** *4 to 6 repetitions per exercise variant*	**Exercise(s)**	**#**
13	37, 38, 39	**Motor Skills Assessment (T3)**				
14	40, 41, 42, 43	Muscle strengthening	Be able to find balance using the tonicity of the body	The child performs the plank exercise by leaning on a Swiss ball. Work in workshops with four different exercises. **Variations**: lean on the elbows or on one foot, move hands on the ground, belly on the ball and feet in suspension.	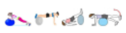	9a
			Be able to perform jumps in different predetermined axes	The child jumps with feet together following the order of the numbers. After each jump, he must return to the center. **Variations**: change the place of the digits.	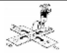	9b
			Be able to perform vertical and horizontal jumps to overcome obstacles	The child jumps sideways over a hurdle with feet together, hands on hips. **Variations**: modify the height of the hurdle, materialize the reception of the feet, first jump over a line drawn on the ground or on a foam roller.	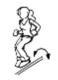	9c
15			Be able to perform lateral jumps and maintain balance on each landing	The child jumps alternately left and right on one foot. **Variations**: modify on the rhythm and the distance between the jumps	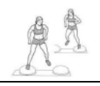	10a
	44, 45, 46	Braking and accelerating sequence	Be able to follow a predefined route with multiple changes of direction	The child must touch each cone and return as quickly as possible to the center mark to make a new start. **Variations**: modify the distance of the cones from the center.	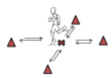	10b
			Be able to move quickly in a sidestep	The child must move sideways, touch the cone on his right and come back to touch the other on his left, passing through the center (four turns). **Variations**: modify the distance between the studs; hold a medicine ball, touch the studs with the feet.	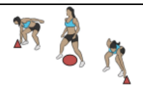	10c
16			Be able to run quickly while controlling your actions	The child must go back and forth to bring the balls from one basket to the other, one ball for each pass. **Variations**: modify the number of balls and the distance between the two baskets.	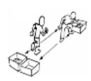	11a
	47, 48, 49	Linear and multidirectional speed with change of direction	Being able to run and change direction quickly	In the form of a contest and on a signal, the children must run as quickly as possible to the plot, go around it and return to the starting line. **Variations**: change the distance	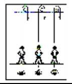	11b
			Be able to run quickly and get around obstacles	Starting in pairs, the child must run the distance as quickly as possible, bypassing the cones and then returning to the starting line. **Variations**: change the distance between obstacles and the overall race distance.	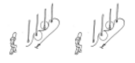	11c
17			Be able to run quickly in a circle with a change of direction	Children form a circle around the teacher and move along the edge of the circle. The teacher announces the change in the direction of rotation which the children must respect quickly. **Variations**: Vary the speed of movement and the diameter of the circle.	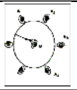	12a
	50, 51	Start from an unusual position	Be able to react quickly to a sound signal from an unusual position	After a sound signal, the child runs from an unusual position: squatting, lying on his back or on his stomach. The exercise is organized in the form of competition. **Variations**: modify the distance to be covered, vary the starting positions, run with a weighted object.	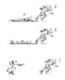	12b
			Be able to run quickly after a jump	The children take turns jumping over a rubber band 40 cm from the ground. They must land with their feet together in a hoop and then continue with a 10 meter run. **Variations**: modify the height of the elastic and modify the reception surface.	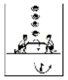	12c
18	52	Review of achievements and reinforcement		All elements already seen in the cycle are resumed in the form of a course	9a, 9b, 9c, 10a	
	53				10b, 10c, 11a, 11b	
	54				11c, 12a, 12b, 12c	
Cool-down routine (5 min) at each session						
19	**Motor Skills Final Assessment (T4)**					
