# Supplementary material for: Impact of a Sensorimotor Integration and Hyperstimulation Program on Global Motor Skills in Moroccan Children With Autism Spectrum Disorder: Exploratory Clinical Quasi-Experimental Study
Source: JMIR Form Res. 2025 Mar 26;9:e65767. doi: 10.2196/65767 (PMC11982752; doi:10.2196/65767)
Supplement: Multimedia Appendix 2 [file formative_v9i1e65767_app2.pdf]

Week	Session	Sequence	Objectif	Exercise description (20 min/exercise including, exercise variations)<br>*4 to 6 repetitions per exercise variant*	Exercises	#
**Cycle 1: Aerobic physical quality: running**						
General (15 min) and specific (5 min) warm-up routine for each session						
1	1, 2, 3	**Motor Skills Assessment (T1)**				
2	4, 5, 6	Aerobic capacity: total volume: 10 to 15 min	Continuous run	The children run into the room while the teacher attempts to hit them with a foam ball. The one who is touched must perform an additional exercise (jump). Children are not allowed to stop **Variations**: alternate brisk walking and running in small strides.	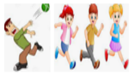	1 a
			Run in different directions	Motor racing: Each child is given a small hula hoop to use as a steering wheel. The children run while the teacher gives them driving instructions. The goal is to keep the children moving in line. **Variations**: drive backwards making periodic stops.	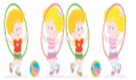	1 b
			Control of a regular speed	The train: the children form two trains by positioning themselves one behind the other, holding each other's shoulders. The train must not detach and follows the direction indicated by the teacher **Variations**: crossing obstacles, holding on to the hips, varying the pace of movement	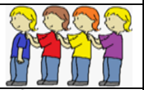	1 c
3	7, 8, 9		Run in a circle with changes of direction	Clock game: The children form a circle around the teacher and move along the edge of the circle. The teacher announces the change in rotational direction that the children must respect. **Variations**: hold hands. Vary the circle's diameter.	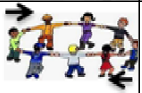	2 a
			Adapt movements according to obstacles	Endurance course: Create a course with different objects that the child must pass under the bench, lie flat on his stomach to crawl, slalom between blocks... The goal is to keep the children moving. **Variations**: Change the layout of the equipment	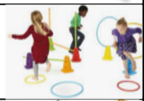	2 b
			Run while varying the pace of movement	A snake formed by children holding each other's shoulders. The head of the snake tries to bite its own tail (the child who is last) which must escape while remaining attached to its friends. **Variations**: change the head of the snake, vary the pace of movement	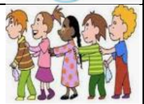	2 c
4	10, 11, 12	Aerobic power: total volume 3 to 6 min. Rep: 1min-45s-30s-20s-15s-10s	Quick side change	Two groups stand face to face in a line. The distance separating the two groups is 5 m. The two groups must change sides quickly at the signal of the teacher. **Variations**: Change starting positions: standing, sitting, lying, on one leg.	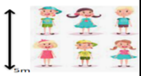	3 a
			Running fast over short distance	A relay game between two teams in single file behind a starting line. At the signal, run towards the wall at a distance of 5 m, touch it, come back and clap on the hand of his friend who does the same thing again. **Variations**: Vary the way of moving	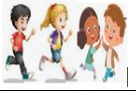	3 b
			Alternate slow and fast pace	The children run to the rhythm of the music which alternates between slow and fast rhythms. **Variations**: drumbeats. Increase the period of the fast rhythm.	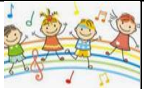	3 c
5	13, 14, 15	Speed: Set: 3 to 4. Rep. 1 to 3	Reaction time	The fox and the shelter: Draw an isosceles triangle with sides of 5 m. The three corners are defined by different colors. The teacher is in the middle announcing a color (shelter) where children must move quickly. The child who is caught is disqualified from the game. **Variations**: Vary the distance between shelters.	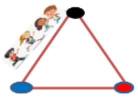	4 a
			Run quickly over short distances	The Crocodile River: Lined up on a starting line, at the signal the children must cross a 6 m wide river and reach the other bank without being caught by the crocodile. **Variations**: Vary the width of the river. Vary signal frequency	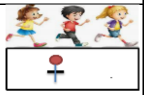	4 b
			Run quickly from an unusual position	Children must run a distance of 6 m as quickly as possible from a different starting position: squatting, lying on their backs, lying on their stomachs… **Variations**: Go back and forth to the starting position.	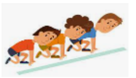	4 c
6	16	Review of achievements and reinforcement		All the elements already seen in the cycle are resumed in the form of a course	1a, 1b, 1c, 2a	
	17				2b, 2c, 3a, 3b	
	18				3c, 4a, 4b, 4c	
Cool-down routine (5 min) at each session						
7	**Motor Skills Assessment (T2)**					

**Cycle 2 : qualité physique force APS support saut/lancer**						
General (15 min) and specific (5 min) warm-up routine for each session						
**Week**	**Session**	**Sequence**	**Objectif**	**Exercise description (20 min/exercise including, exercise variations)** *4 to 6 repetitions per exercise variant*	**Exercises**	**#**
8	19, 20, 21					
9	22, 23, 24	Horizontal jump (explosive strength)	Run with changes of direction	After a run-up, cross rivers following the direction of the arrows. **Variations**: Width of rivers, number of passages, number of obstacles.	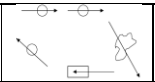	5 a
			Run-up and jump connection	After a run-up of three or four steps, take off on an elevated surface (plinth, step) and land on a mat with two feet. **Variations**: Height of the elevated surface, mark the areas to be crossed during the jump with adhesive tape.	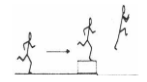	5 b
			Direct the jump forward	After the take-off, the child must reach the furthest area drawn on the mat (four areas). Game in the form of competition. **Variations**: Change the take-off distance.	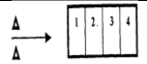	5 c
10	25, 26, 27	Vertical jump (explosive strength)	Direct the jump upwards by mobilizing your hands	Students must jump to touch hanging objects with their hands. Allow at least 5 passages per object. **Variations**: Vary the height of objects. Jump with or without a backswing.	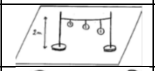	6 a
			Direct the jump upwards.	“The hock cutter”: Students must cross a stretched rubber band, moved by two people, 20 cm from the ground. **Variations**: The speed of movement of the rubber band, the direction of movement, the height.	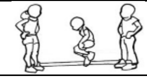	6 b
			Run-up and impulse connection	After a run-up and a take-off, jump to an elevated surface and land on both balanced feet. **Variations**: Vary the platform height.	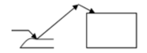	6 c
	28, 29, 30	Organisation ses actions pour lancer un objet	Make as many throws as possible with different objects	“The burning ball”: Two groups face each other separated by benches. The goal is to throw objects of different textures and weights towards the opposing camp **Variations**: Vary the height that separates the two camps and the surface of the field	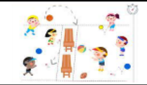	7 a
11			Throw at a stationary target	From a determined area, throw objects of different textures and weights to hit as many targets as possible (weighted balls, sandbags or tennis balls. **Variations**: Vary the distance of the throw and the weight of the objects.	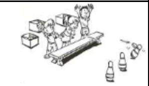	7 b
			Throw at a moving target	“Game hunters”: Two teams facing each other: Hunters try to hit the running game with their foam balls. **Variations**: Switch roles, change throwing distance.	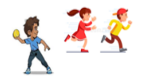	7 c
	31, 32,33	Orientation et maitrise du lancer	Throw at a vertical target	The child throws from three throwing areas, different sandbags to hit a vertical target. **Variations**: Vary the height and diameter of the target, the weight of the sandbags.	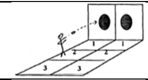	8 a
12			Throw at a horizontal target	The child throws sandbags from three different throwing areas to hit a horizontal target one meter in diameter. **Variations**: Modify the diameter of the target and the weight of the sandbags.	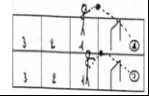	8 b
			Throw a ball over a horizontal obstacle	The child tries to reach the area as far as possible by throwing a medicine ball over a 1.50 m high net. Variants: Medicine ball of different weight and volume. Change the height of the net.	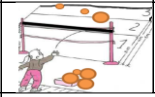	8 c
13	34	Review of achievements and reinforcement		All the elements already seen in the cycle were resumed in the form of a course.	5a, 5b, 5c, 6a	
	35				6b, 6c, 7a, 7b	
	36				7c, 8a, 8b, 8c	
Cool-down routine (5 min) at each session						
14	**Motor Skills Assessment (T3)**					

Cycle 3: Football/Basketball cooperation and opposition						
General (15 min) and specific (5 min) warm-up routine for each session						
**Week**	**Session**	**Sequence**	**Objectif**	**Exercise description (20 min/exercise including, exercise variations)**<br>*4 to 6 repetitions per exercise variant*	**Exercises**	**#**
15	37, 38, 39					
16	40, 41, 42, 43	Exchange and social interactions	Awareness of a partner	The children form a circle. A child passes a ball to another child in front of him. Then the child does the same and so on until the ball has passed through all hands. **Variations**: Change the direction of the ball.	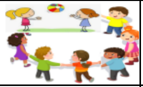	9a
			Participate in a game with others	"Ball in cage": The children are arranged in a circle and roll the ball between them without leaving the circle. **Variations**: Exchange the ball by passing with the feet (foot). Modify the diameter of the circle.	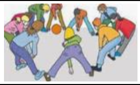	9b
			Take on a task within a group	"The chain of firefighters": The children are each lined up in a hoop and pass balloons to empty the red box and fill the green box. **Variations**: The hoops are spaced out, change the location of the children.	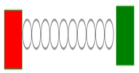	9c
17			Participate in a collective task	Children stand in a circle and try to keep an inflated balloon in the air for as long as possible. **Variations**: Play in a small group. Introduce two then three balloons at the same time.	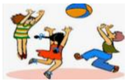	10a
	44, 45, 46, 47, 48	Discover a collective game	Play in a visually delimited space	Children are standing inside a circle materialized by studs, and try to maintain an inflated balloon in the air, as long as possible. Variation: Play on a rectangular court with a soccer ball and/or a basketball.	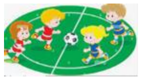	10b
			Integrating the notion of target into a game space	Playing 3 against 3 on a half court with a target to attack which directs the direction of the game. **Variations**: Ambush with objects to drop and hoop. Introduce a target to defend.	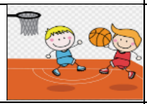	10c
			Discover 3 technical gestures in football (driving, passing, shooting a ball)	In limited space children shoot pins, dribble the ball down a roped lane and pass the ball up the wall. **Variations**: Modify the distance of shooting, passing and dribbling the ball.	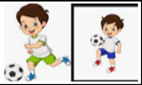	11a
			Discover 3 technical gestures in basketball (throw, receive, dribble)	With one ball per child in a restricted space, throw in the air and catch the ball, dibble and shoot. **Variations**: Restrict the child's mobility in a 1 meter-wide diameter hoop.	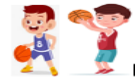	11b
18			Understand and respect the rules during a game	3X3 match in two sequences of games: Foot: Attention is paid to the hand fault. In basketball, attention is paid to the fault of violent contact. **Variations**: Other faults will be introduced progressively. Avoid using the whistle.	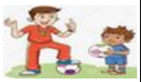	11c<br>12a
	49,50, 51	Differentiate between partner and adversary	Pass-receive while avoiding an obstacle	Two by two, pass and receive a ball around a fixed obstacle (dummy or cone). **Variations**: Modify the distance between the children. Alternate Football and Basketball	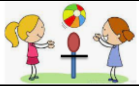	12b
			Progress over a distance around obstacles	Make a progression with two players over a distance of 6 m around fixed obstacles (dummies, cones) without dropping the ball. **Variations**: Vary the distance of progression.	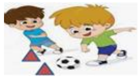	12c
			Progressing in the presence of an opponent with the ball and shooting at a target	Advance in pairs in the presence of a defender over a distance of 6 m and throw to the basket. **Variations**: Advance to three in the presence of a passive then active defender.	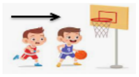	
19	52	Review of achievements and reinforcement		All the elements already seen in the cycle were resumed in the form of a course.	9a, 9b, 9c, 10a	
	53				10b, 10c, 11a, 11b	
	54				11c, 12a, 12b, 12c	
Cool-down routine (5 min) at each session						
20	**Motor Skills Final Assessment (T4)**					
